# Identification of SCRG1 as a Potential Therapeutic Target for Human Synovial Inflammation

**DOI:** 10.3389/fimmu.2022.893301

**Published:** 2022-05-26

**Authors:** Guoqiang Liu, Guisong He, Jie Zhang, Zhongmin Zhang, Liang Wang

**Affiliations:** ^1^ Department of Orthopedics, Academy of Orthopedics, The Third Affiliated Hospital, Southern Medical University, Guangzhou, China; ^2^ Department of Orthopedics, The Second Affiliated Hospital, Guangzhou Medical University, Guangzhou, China; ^3^ Division of Spine Surgery, Department of Orthopedics, Nanfang Hospital, Southern Medical University, Guangzhou, China

**Keywords:** osteoarthritis, synovium, SCRG1, LASSO, pharmaceutical therapy

## Abstract

Synovial inflammation of joint tissue is the most important cause of tissue damage, joint destruction, and disability and is associated with higher morbidity or mortality. Therefore, this study aims to identify key genes in osteoarthritis synovitis tissue to increase our understanding of the underlying mechanisms of osteoarthritis and identify new therapeutic targets. Five GEO datasets with a total of 41 normal synovial membrane tissues and 45 osteoarthritis synovial membrane samples were used for analysis, and seven common differential genes were identified. The classification model constructed by LASSO analysis showed that six genes including CDKN1A, FOSB, STMN2, SLC2A3, TAC, and SCRG1 can be used as biomarkers of osteoarthritis, and the SCRG1 gene shows importance in osteoarthritis. Furthermore, drug database enrichment found that these six DEGs may be the drug targets of synovitis in osteoarthritis, and Valproic Acid CTD 00006977 may be a potential targeted therapeutic drug of SCRG1. Spearman correlation analysis was performed on the SCRG1 gene, and 27 genes with consistent expression were obtained. Functional analysis showed that 27 genes were mainly involved in metabolism, complement, antigen presentation, apoptosis, and regulation of immune pathways. The co-regulatory network of TFs-miRNA suggested that the SCRG1 gene may be regulated by hsa-miR-363-3p miRNA. In conclusion, SCRG1, as a diagnostic marker of osteoarthritis, co-regulates immune-related pathways through the interaction of related proteins, playing an important role in the occurrence and development of osteoarthritis, which may be a novel drug target.

## Introduction

Osteoarthritis (OA) is one of the most common joint diseases and the main cause of disability in the elderly, accounting for 30% to 50% of people over 65 years old. The occurrence of OA is related to aging, obesity, inflammation, trauma, overuse of joints, metabolic disorders, and genetic factors (1, 2). OA is characterized by articular cartilage injury and degeneration, accompanied by subchondral bone sclerosis, hyperplasia, and cystic degeneration, followed by joint space narrowing, meniscus, and synovitis. The typical symptoms of OA include pain, swelling, and stiffness, often accompanied by dysfunction and limited movement. Its clinical treatment includes pain relief and slowing tissue degradation (3–5). However, currently, there is no effective drug for treatment that can slow down the progression of the disease, as the exact pathogenesis of OA is still unclear (6–11).

Synovium is the boundary between an internal structure of the joint and the adjacent soft tissue, which is very important to maintain the stability of the internal environment. Although the main histopathological changes in osteoarthritic joints are cartilage destruction and chondrocyte proliferative differentiation, synovial lesions are also clinically found in many joint diseases and may play an important role in promoting the development and progression of the disease (12). In previous studies, it has been found that OA-related synovitis is a cartilage matrix degradation product caused by the secondary change. Synovitis is not a bystander in the occurrence of OA, but a participant in the destruction of joint structure, which promotes the progression of OA. Therefore, synovium may be a potential target for the treatment of OA (13, 14).

The pathogenesis of OA is complicated. It has been reported that a variety of inflammatory factors, including adipokine, interleukin, nerve growth factor, and tumor necrosis factor, can affect the progression of OA (14–16). In recent years, high-throughput chip technology has made great progress in molecular diagnosis and classification, prognosis prediction, target drug discovery, and other fields of OA research. Some gene expression profile studies on OA have revealed several key genes, diagnostic gene biomarkers, and related signaling pathways. Recent studies have implicated that secreted damage-associated molecular patterns (DAMPs) can act as ligands of Toll-like receptors (TLRs) or receptors for advanced glycation end-products (RAGEs) influencing synovial inflammation (17, 18). Impact injury stimulates the release of reactive oxygen species (ROS) that induce cell death in OA and activation of stress-induced kinases that upregulate MMP-13, ADAMTS-5, and TNF-α (19, 20). Other research reports that NOS-2, COX-2, MMPs, and ADAM were also associated with OA (21). In MicroRNAs, Tegner J et al. found in the OA FLS that the increased levels of miR-625 and miR-124 are related to the decreased expression of their target genes, while the expression levels of miR-155b and miR-203 are lower and the expression of their target genes is higher (22). Wang Q et al. found that NEAT1/miR-181c can regulate the proliferation of synovial inflammatory cells in OA (23). Similarly, inflammatory activation of the synovium can lead to the release of various proinflammatory mediators that not only cause widespread changes in the structure and function of synovial tissue but also promote articular cartilage damage and accelerate OA development (13). As several studies have shown, the release of inflammatory cytokine IL-1 β can induce and activate multiple immune signaling pathways including NF-κB, PI3K/AKT and MAPK, and then collagenase such as matrix metalloproteinase-13 (MMP-13) degradation of the extracellular matrix (24, 25). The disruption of the wnt signaling pathway is also associated with the pathogenesis of OA (26). Although there have been several studies on OA and new therapies that may help maintain joint homeostasis, little effect has been achieved (7, 11). Therefore, the biological treatment of inflammatory factors or intracellular inflammatory signal molecules is still the research direction of OA treatment. The research on the pathogenesis of OA and related genes still needs to be further studied. If potential diagnostic molecular markers of OA can be screened out before the onset, the quality of life of patients can be improved. In addition, biomarkers can help identify the early degradation of OA and may be applied to clinical practice decisions.

In view of this, in order to describe OA at the molecular level and reveal its pathogenesis and find suitable targets, we attempted to find differentially expressed genes (DEGs) in osteoarthritis by obtaining similar synovitis data for integrated analysis. We made use of the LASSO machine learning method to identify the key biomarkers and used the drug database and function enrichment method to obtain the ideal drug targets for osteoarthritis.

## Materials and Methods

### Collection of the Dataset

The dataset GSE1919, GSE12021, GSE55235, GSE55457, GSE82107, which is based on the Affymetrix Human Genome Array platform, and dataset GSE89408, GSE143514, which is based on Illumina HiSeq 2000 were downloaded from the Gene Expression Omnibus (GEO) database (http://www.ncbi.nlm.nih.gov/geo/). All selected datasets were genome-wide expression data in joint synovial membrane tissues of OA or normal synovial membrane tissues. For all samples used in this study, the disease group included synovial membrane samples from patients who had undergone open synovectomy, arthroplasty, or joint replacement, and the normal control group included synovial membrane samples from patients who had undergone fatal accidents or joint trauma surgery, postmortem, or traumatic joint injury. Healthy subjects were defined as those who had no evidence of any form of arthritis on history or examination and had no cartilage damage or synovitis on knee arthroscopy. OA patients according to the respective criteria for OA and all patients suffered from synovitis. All the samples used above were obtained with the informed consent of patients or their families, and the study of each GEO dataset was approved by their respective Ethics Committee. In total, we obtained 41 normal and 45 OA human synovial membrane tissues in Array data and obtained 27 normal and 31 synovial membrane tissues in RNA-seq data. All datasets were generated by different laboratories. Because of the different sources of data production, we only used chip Array data for further analysis and used RNA-seq data to verify whether the expression of biomarkers in different tissues is consistent.

### Common Gene Identification Between Synovial and Normal Tissues

Identification of DEGs for Array datasets is the primary task of this research. For microarray data, “GEOquery” and “limma” R software package was used to perform the differential gene expression analysis of each dataset between synovial and normal knee cartilage tissues, and P-value were adjusted using the Benjamini and Hochberg method. In brief, the microarray matrix data were quartile data normalization and probe summarization was performed by the Robust Multi-array Average (RMA) algorithm. For RNA-Seq data, differential expression analysis was performed using Deseq2. In this study, genes with |log2fold change (FC) | > 2 and P-value < 0.05 between two groups were considered significantly different. The DEGs of each data set were annotated with Entrez IDs, official gene symbols, and gene names, and the intersection of DEGs between the synovial membrane and blood samples was shown by the Venn diagram and upset plot. During validation with microRNA GEO dataset, expression profiles obtained from the GEO dataset were analyzed with DEseq2, and the genes with a P-value less than 0.05 were considered differential expression in the GEO dataset. For Spearman correlation analysis, datasets from different research of synovial tissue were using limma and sva R software packages to remove batch effect and normalization, the associations between DEGs were evaluated by the Pearson correlation coefficient and Kruskal-Wallis test. All the analyses were performed in R (v4.0.2) using various bioconductor packages.

### Identification of the Optimal Diagnostic Gene Biomarkers for OA

Least absolute shrinkage and selection operator (LASSO) has a strong predictive value and low correlation and is applied to select the best features for high-dimensional data. In order to identify optimal diagnostic gene biomarkers for OA, we utilized the seven key DEGs obtained between synovial and normal tissue from all microarray datasets as feature variables to construct the LASSO model. The LASSO algorithm analysis was performed by the ‘glmnet’ R software package to reduce data dimensions. A model index for each sample was created using the regression coefficients from the LASSO analysis to weight the expression value of the selected genes with the following formula: Index = ExpGene1*Coef1 + ExpGene2*Coef2 + ExpGene3*Coef3+… The “Coef” is the regression coefficient of the gene and is derived from the LASSO Cox regression, and “Exp” indicates the expression values of the gene. Then, five microarray datasets were randomly assigned to the training set (70%) and test set (30%). In order to evaluate the diagnostic ability of the above models and each gene biomarker to identify OA, we evaluated the receiver operating characteristic (ROC) area under the curve (AUC), sensitivity and specificity.

### Gene Ontology and Pathway Enrichment Analysis

For the interest gene sets, Goseq (version 2.0), Kobas (version 3.0), and clusterProfiler (version 4.0) were used for GO and KEGG pathway enrichment analysis. P-value less than 0.05 was set as the cut-off criterion for the significant functional enrichment. Fisher’s exact test (two-side) was performed to classify the pathway category. The false discovery rate (FDR) was used for the P-value correction. “Cluogo” plug-in based on Cytoscape software was used for network visualization of the enriched pathways. Gene set enrichment analyses (GSVA) were performed by the “gsva” R software package to identify enriched immune-related signaling pathways in OA. Spearman correlation analyses and linear regression analyses were performed for the correlation between genes or pathways. For the Gene Set Overrepresentation test, pathways with a false discovery rate less than 0.05 were considered significantly enriched.

### Candidate Drugs Identification

Drug molecule identification is the key component of the ongoing research and molecular analysis of the key target gene can reveal the pathway of drug targeting. To better assist researchers in drug design, we perform drug analysis of DEGs using EnrichR (https://amp.pharm.mssm.edu/Enrichr/) analysis platform and DsigDB database. Each drug molecule in the platform contains the P-value and binding capacity of targeted genes, and the threshold value of adjusted P-value less than 0.05 as a significant result. The EnrichR platform also contains numerous analysis modules, including functional analysis, miRNA targeting analysis, and more.

### Protein-Protein Interaction Network Construction and Module Analysis

In order to understand the associations between proteins of related genes, we used STRING database (version 11.0) integrating functional interactions from known and predicted protein-protein association data to predict the protein interaction network and function. For sub-network analysis, the Molecular Complex Detection (MCODE) plugin tool under the Cytoscape software package was used to cluster the large PPI network into smaller networks. Then Cytoscape software was used to visualize the network. The interactions between gene sets with a combined score > 0.9 were considered to be a key gene.

### TF-miRNA Co-Regulatory Network

TFs-miRNA co-regulatory network can better understand the development mechanism of disease. In this research. NetworkAnalyst (https://www.networkanalyst.ca/) analysis platform is used to identify TF-miRNA interaction with identified common genes. NetworkAnalyst is a comprehensive web platform for performing gene expression network analysis, and the interaction network is obtained from the ENCODE (https://www.encodeproject.org/) database. Cytoscape was used to visualize the NetworkAnalyst analysis results.

### Statistical Analysis

All statistical tests performed were two-sided using the R software by version 4.0.2 (http://www.R-project.org/). For two groups, unpaired Wilcox-test was used to measure the difference of continuous variables between normal groups and OA groups.

## Results

### Identification of DEGs in OA Synovial Membrane

**Figure 1:**
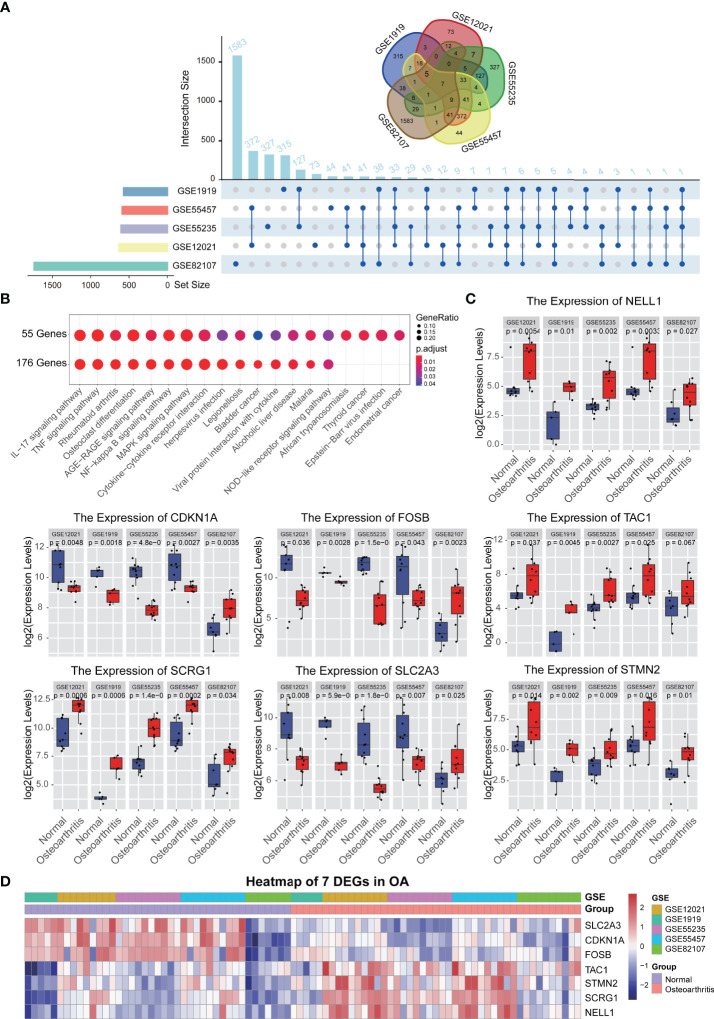
Differential expression gene analysis. **(A)** The different analysis results of 5 synovium-related GEO data sets, upset and Venn diagram showed the relationship between the number of different genes in different GEO datasets. **(B)** KEGG function analysis of each gene sets. **(C)** Boxplot showed the expression levels of 7 genes which are commonly differentially expressed between synovial tissues and normal tissues in 5 GEO datasets. **(D)** A heatmap plot of 7 common differentially expressed genes in all synovial tissues and normal tissues.

The purpose of this study was to use relevant differentially expressed genes (DEGs) in synovial membrane samples to identify dysregulated genes and pathways and to better explain the pathogenesis of OA disease. To achieve this purpose, five GEO datasets (GSE1919, GSE12021, GSE55235, GSE55457, GSE82107) were included in our study as shown in **Table 1**. Then we performed differential analysis, a total of 3118 DEGs were identified in the OA datasets compared with normal tissues, including 293, 188, 287, 191, 289 up-regulated and 270, 419, 218, 375, 1146 down-regulated DEGs in five GEO datasets, respectively. To identify common DEGs in OA, we performed an integrative analysis, a total of 176 DEGs overlapped in 3/5 datasets, a total of 55 DEGs overlapped in 4/5 datasets, and a total of seven DEGs were obtained in five datasets (**Figure 1A**, **Table S1**). Furthermore, we used KEGG to analyze the functions of each gene set; the analysis results showed that both 176 and 56 DEGs were enriched immune-related pathways, such as Rheumatoid arthritis pathway, IL-17 signaling pathway, TNF signaling pathway, NF-kappa B signaling pathway, MAPK signaling pathway (**Figure 1B**). On the other hand, we also analyzed these seven DEGs, and the results showed that seven genes were significantly expressed in normal and synovial samples (**Figure 1C**), hierarchical clustering analysis of the seven DEGs was presented in **Figure 1D** and gene description with gene functions is shown in **Table S1**. These genes and related pathways analyzed above may be associated with the pathogenesis of OA.

**Table 1:** Datasets of gene expression profiles.

ID	GEO	Sample source	OA samplessamples	Healthy samplessamp	Seq Type	Platform	year	country	author
1	GSE1919	Synovial tissue	5	5	Array	[HG_U95A] Affymetrix Human Genome U95A Array	2004	Germany	Ute Ungethuem
2	GSE12021	Synovial tissue	10	9	Array	GPL96[HG-U133A] Affymetrix Human Genome U133A Array	2008	Germany	Huber R
3	GSE55235	Synovial tissue	10	10	Array	GPL96[HG-U133A] Affymetrix Human Genome U133A Array	2014	Germany	Woetzel D
4	GSE55457	Synovial tissue	10	10	Array	GPL96[HG-U133A] Affymetrix Human Genome U133A Array	2014	Germany	Woetzel D
5	GSE82107	Synovial tissue	10	7	Array	GPL570 [HG-U133_Plus_2] Affymetrix Human Genome U133 Plus 2.0 Array	2019	Netherlands	de Vries M
6	GSE89408	Synovial tissue	22	28	RNA-Seq	Illumina HiSeq 2000 (Homo sapiens)	2016	USA	Alice Walsh
7	GSE143514	Synovial tissue	5	3	RNA-Seq	Illumina HiSeq X Ten (Homo sapiens)	2020	China	Zhou Y

### Identification of the Optimal Diagnostic Gene Biomarkers for OA

**Figure 2:**
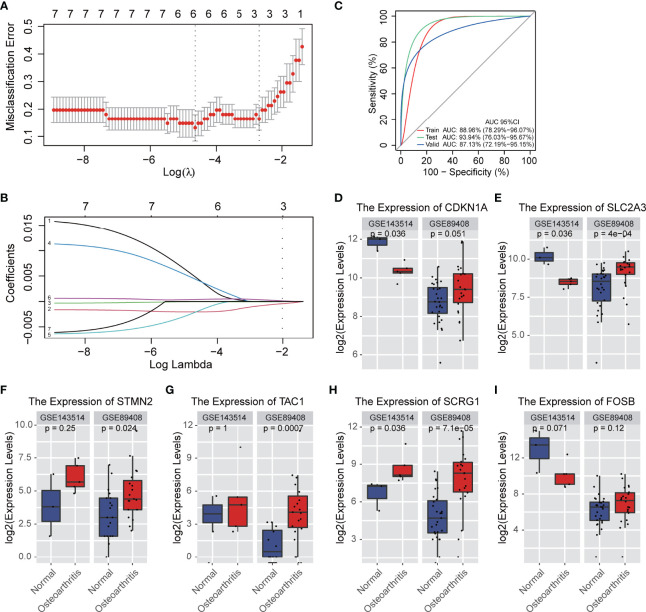
A model for predicting OA and verification of gene differential expression. **(A)** CV statistical graph during the construction of the LASSO regression model, which shows that the minimum lambda at model construction is 0.00154252 (dotted line on the left). **(B)** the model regression coefficient diagram shows the change trend of the coefficient corresponding to each gene variable with the change of lambda value. The results showed that there were 6 prediction genes corresponding to the minimum lambda. **(C)** ROC curve predicts the identification effect of the above models in different datasets. The closer AUC value is to 1, the better of prediction effect on the model. The figure is shown that the AUC in the training set is 0.8896 and that in the test set is 0.9394, indicating that the model has good prediction accuracy. In RNA-seq validation data set, the AUC is 0.8713, which shows that the models constructed by the 6 genes can also have good accuracy in different types of data sets. **(D–I)** The expression levels of 6 genes affecting disease occurrence in the model are verified. The figure shows that 6 genes are also significantly differentially expressed in the RNA-SEQ data set.

In order to determine the best diagnostic gene biomarkers for OA, we extracted the expression profile of seven DEGs that are commonly differentially expressed from five GEO datasets to construct the LASSO model (**Figure 2A**). First, we divided the five GEO dataset samples into training sets and test sets in a ratio of 7:3, and then performed LASSO regression analysis. Using the LASSO method, seven DEGs were identified with non-zero regression coefficients, and the value of lambda.min was 0.00154252, according the threshold value, gene Nell1 excluded (**Figure 2B**). The six DEGs-based model index was created as the following formula: Index=TAC*(0.0051) + CDKN1A*(-0.002) + FOSB*(-0.001) + STMN2*(0.0052) + SLC2A3*(-0.0014) + SCRG1*(0.0004). Therefore, we selected these six DEGs as the optimal potential diagnostic gene biomarkers for OA. ROC curve analysis showed that the AUC of the six DEGs-based model was 0.8896 (95% CI: 78.29%-96.07%) in the training set and 0.9394 (95% CI: 76.03%-95.67%) in the test set; this result indicated the LASSO model has a high AUC value and may serve as a better biomarker of OA (**Figure 2C**). In addition, two RNA-seq GEO datasets (GSE89408 and GSE143514) associated with synovial membrane were used as validation sets to verify the accuracy (**Figure 2C**); the validated AUC value is 0.8713 (95% CI: 72.19%-95.15%). This is also a high accuracy to predict the occurrence of OA. Furthermore, we examined the six DEGs expression in two RNA-seq datasets and we found that SCRG1, CDKN1A, and SLC2A3 are significantly differentially expressed in OA which has the same expression trend with five microarray data (P-value<0.05), and indicated that these three DEGs were highly associated with OA, and they could play an important role in disease (**Figures 2D–I**).

### Identification of Candidate Drugs for Six DEGs

Previous differential analysis and biomarker studies showed that six DEGs (TAC, CDKN1A, FOSB, STMN2, SLC2A3, SCRG1) play an important role in OA and may serve as disease targets, so we used EnrichR platform to analyze drug molecules that may be involved in the six genes. The drug data were collected from the DSigDB database. According to the adjusted P-value, the results from the candidate drugs were generated (**Table S2**). The drug result showed that there were 100 possible drug molecules targeting six DEGs, of which VALPROIC ACID CTD 00006977 was the common drug molecule associated with six DEGs, Tetradioxin CTD 00006848, Retinoic acid CTD 00006918, estradiol CTD 00005920, benzo pyrene CTD 00005488, and Silica CTD 00006678 were also significant drug molecules that interacted with other DEGs. Interestingly, we found that Retinoic acid-related orphan receptor (RORα) is a protein family that expressed in a variety of tissues such as retina, skeletal muscle, brain, thymus, liver, lung, kidney, and adipose tissue, and may mediate the process of OA by alterations in cholesterol metabolism (27). The inverse agonist of the retinoic acid-related orphan receptor has shown the potential to modify the progression of OA with minimal adverse effects (28). Combined with the results obtained in this study, it is suggested that the four DEGs (CDKN1A, FOSB, SLC2A3, SCRG1) may be the target of retinoic acid drugs, and other drugs in the list may also be targets for drug design in OA disease.

### Spearman Correlation Analysis Suggested the Function of the SCRG1 Gene

For the six marker genes previously identified, we found that SCRG1, CDKN1A, SLC2A3 genes were significantly expressed in all microarray and RNA-seq datasets, and only SCRG1 gene showed an increased expression trend in all datasets. SCRG1 has also been observed to be specifically expressed in human articular cartilage (29–31), so we considered that SCRG1 might be a key regulatory factor in the development of synovial inflammation.

**Figure 3:**
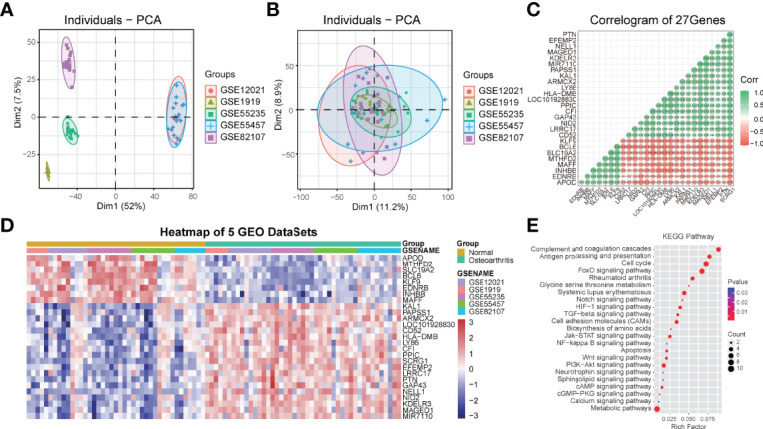
PCA cluster plot before and after sample correction and protein-protein interaction analysis of spearman correlation genes with SCRG1. **(A, B)** Two-dimensional PCA cluster plot of the 5 microarray datasets before and after sample correction, the colors represent each osteoarthritis datasets. **(C)** The Spearman’s correlation coefficients of gene expression with SCRG1, 27 genes were found. **(D)** Heatmap plot of 27 associated genes, and showed 27 genes also differentially expressed in all synovial tissues and normal tissues. **(E)** KEGG pathway analysis of the 27 genes, and the enriched immune-related pathways were shown.

In order to understand the role of SCRG1 in OA, we performed gene expression correlation analysis to predict SCRG1 gene function by protein-protein interaction. First, we removed the batch effect and normalized it from the gene expression matrix after merging five microarray datasets, and PCA analysis was used to show the effect (**Figures 3A, B**). The PCA result showed that the cluster of each dataset sample was more concentrated, indicating that the batch difference has been removed and the sample matrix was reliable. Then, Spearman correlation analysis was performed to estimate the correlation of the SCRG1 gene with other genes at the expression level in the normalized data. The Spearman analysis results showed that 27 correlation genes were obtained, and these genes expressions were also differential in each microarray dataset (**Figures 3C, D**).

To ascertain the possible mechanisms of SCRG1 gene affecting OA progression, GO function and KEGG pathway analysis were used. The most significantly enriched GO terms based on the 27 genes in the biological process category were signal transduction, in the cellular component category was MHC protein complex, and in the molecular function category was receptor activity (**Figures S1A–C**). These results are consistent with previous research. For example, Mohammadi et al. found that aberrant signaling contributes to the maldevelopment of joints and the onset and progression of OA (32), and TLRs receptors or ligands also affect synovial membrane inflammation (17, 19).

The KEGG pathway analysis result showed some pathways like complement and coagulation cascades, antigen processing and presentation, cell cycle, rheumatoid arthritis, cell adhesion molecules (CAMs), metabolic pathways, FoxO signaling pathway, PI3K-Akt signaling pathway, Jak-STAT signaling pathway, TGF-beta signaling pathway, Wnt signaling pathway, Notch signaling pathway, NF-kappa B signaling pathway, apoptosis, and other signaling pathways were all quite significant in key cascades in the basic biology of OA, such as initiation, growth, maintenance, and development of OA (**Figure 3E**). Interestingly, “MAPK signaling pathway,” “PI3K-Akt signaling pathway,” “NF-kB signaling pathway,” “TGF-beta signaling pathway,” and “Wnt signaling pathway” have been reported to be essential for OA-immune evasion in human cartilage tissue (33–37), but these pathways were rarely reported in synovium with OA, and these pathways are also involved in other specific immuno-associated disease, such as in RA, SLE, and AS, many of which have shown optimistic response on therapy. Moreover, many other pathways were also found to be associated with OA in our results, such as cell adhesion molecules (CAMs), metabolic pathways, complement and coagulation cascades, antigen processing and presentation, apoptosis, and cell cycle.

### PPIs Network Analysis of Correlation Genes to Identify Potential Molecular Signatures in Osteoarthritis Synovium

**Figure 4:**
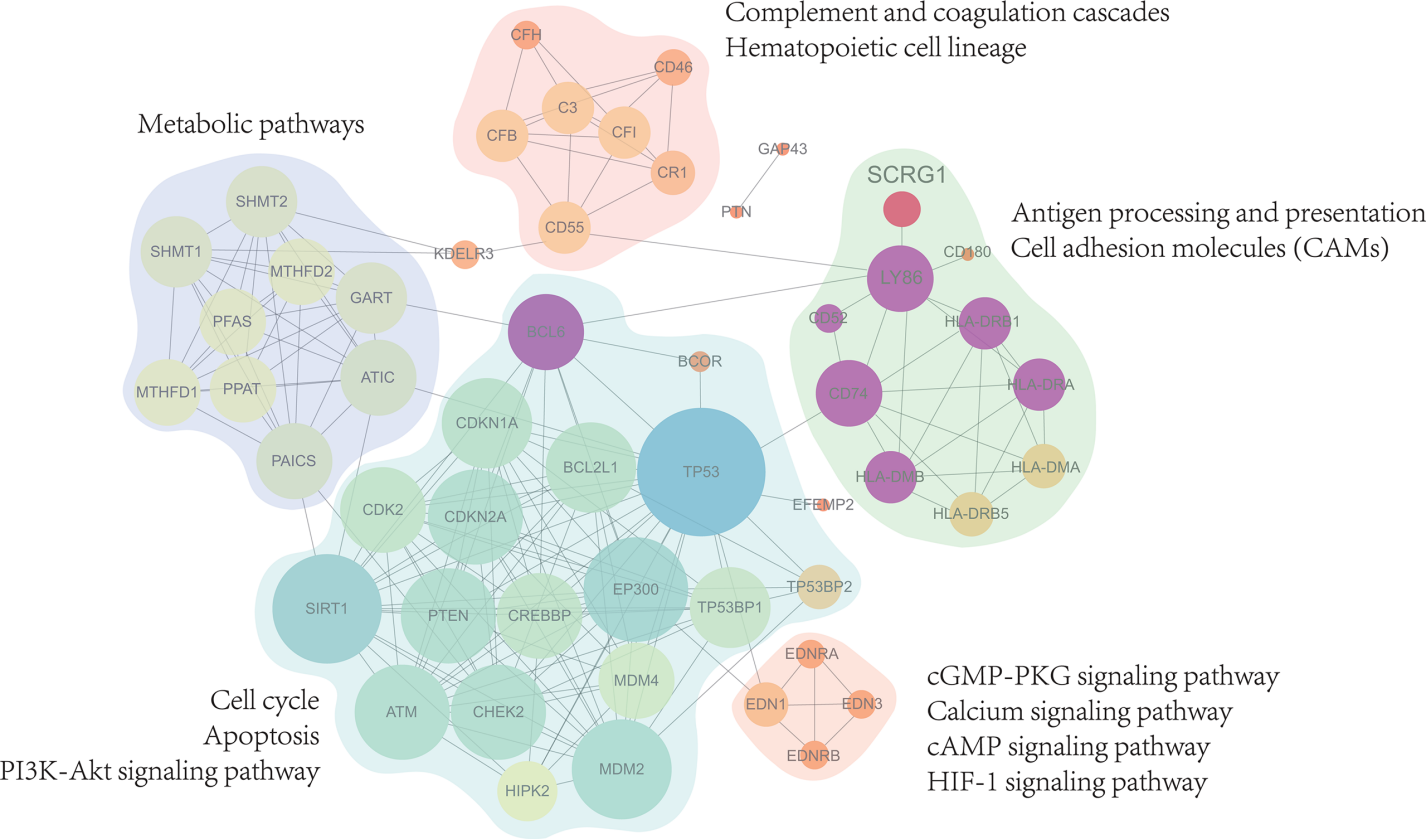
Protein-Protein interactions analysis with 27 genes associated expression with SCRG1. The PPI network showed 5 sub-functional clusters were identified and suggest that SCRG1 may be a protein complexes service with important biological function in OA. And the biological function is mainly related to metabolic pathways, complement and coagulation cascades, Antigen processing and presentation, Apoptosis, and immune-related signaling pathway.

To improve the biological understanding of the correlation between 27 gene functions identified in this study, we conducted protein-protein interaction analysis. As shown in **Figure 4**, the protein-protein interactions result shows that 27 protein interaction network was divided into five subnetworks, molecular functions of the five subnetworks are mainly related to metabolic pathways, complement and coagulation cascades, antigen processing and presentation, apoptosis, and immune-related signaling pathway, respectively. Those subnetwork results suggest that the similar functional proteins may be protein complexes serving important biological functions in OA, which are closely associated not only with the growth process of OA but also with the synovial membrane environment. Furthermore, in this part of the result, we found that SCRG1 gene only participates in one subnetwork and serves immune-related biological functions such as antigen processing and presentation. On the other hand, SCRG1 only interacts with Ly86 gene, which may be due to the lack of research on the SCRG1 gene. For other functions in the PPIs network, we have reason to suspect that SCRG1 may interact with LY68 and form protein complexes with other proteins to perform corresponding functions. Therefore, SCRG1 as a potential predictive marker needs to be further investigated.

### Co-Regulatory Network Mapping and Dysregulated Targets of Regulated miRNA

**Figure 5:**
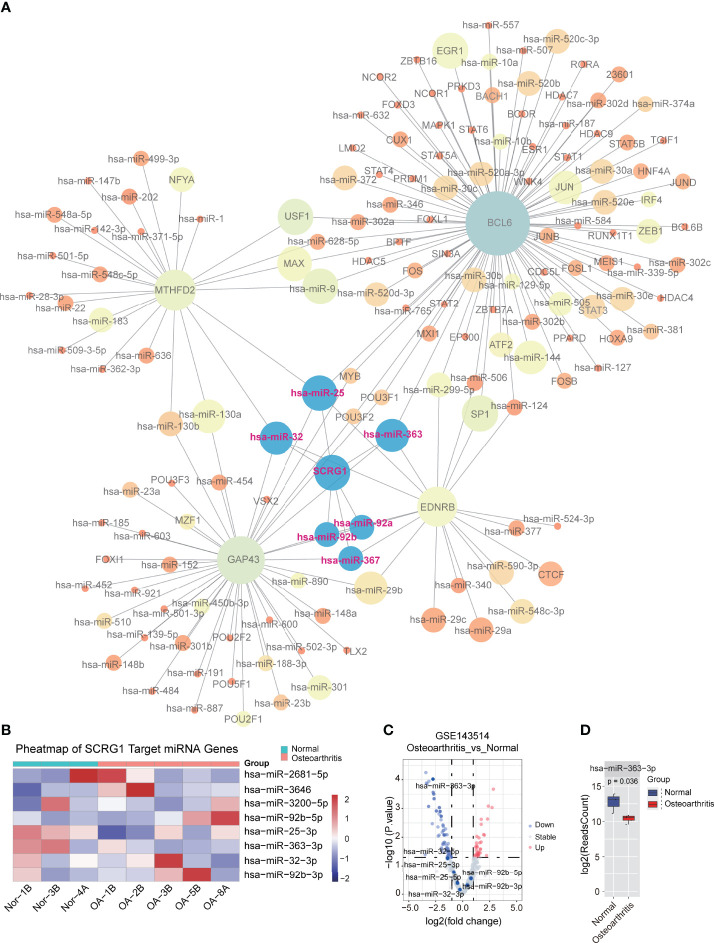
TF-miRNA co-regulatory network of 27 genes associated with SCRG1. **(A)** The TF-miRNA co-regulatory network for 27 genes, SCRG1 was not found as the corresponding transcript factor in network, but 6 miRNAs can regulate SCRG1 gene expression. **(B,C)** volcano and heatmap plot of the miRNA expression in GSE143514 dataset, which represents the differential expression of values in synovium and normal tissues. Red color dot in Volcano plot represents up-regulated expression in synovium tissue. **(D)** hsa-miRA-363-3p was significantly differentially expressed in synovium tissues.

The analysis of the TF-miRNA co-regulatory network delivers miRNAs and TFs interaction with the common DEGs. This interaction can be the reason for regulating the expression of the DEGs. The regulatory network between 27 correlated genes and TFs, miRNAs were constructed using NetworkAnalyst that consisted of 657 edges and 432 nodes between 142 TFs, 261 miRNAs with 27 correlation genes in the context of OA (**Figure 5A**). The constructed regulatory network showed that four genes (BCL6, MTHFD2, GAP43, EDNRB) were high TFs and miRNAs interactions. In addition, multiple TFs that regulate these four genes representing differential expression in OA were identified as crucial TFs: JUN, SATA2, FOS, TP53, and MYC, which are associated with the development of OA. These TF-genes regulate more than one common correlation gene of the network, which indicates high interaction of the TF-genes with common genes. Interestingly, we found that six miRNAs regulate SCRG1 gene, and no TFs associated with SCRG1 gene in the regulatory network. To confirm the regulatory results of miRNA, miRNA sequencing data from GSE143514 were used to validate the miRNA expression level between the synovial membrane and normal tissue. We use DEseq2 method for miRNA differential expression analysis, and the volcano plot showed the differential expression of six miRNAs. The results showed hsa-miR-363-3p was significantly expressed and P-value of 0.00095 (**Figure 5C**), and heatmap showed six miRNAs were changed by a different expression level, indicating that six miRNAs, especially hsa-miR-363-3p, may play an important role in OA (**Figures 5B, D**). The SCRG1 gene may be regulated by multiple TFs in OA, affect the expression of targeted miRNA, and regulate the functions of other disease genes, which may play a critical role in the pathogenesis of OA and be a potential therapeutic target.

## Discussion

Osteoarthritis is a chronic joint disease with an increasing prevalence and burden of cartilage degeneration. The disease will cause cartilage degeneration, synovial inflammation, subchondral bone remodeling, and osteophyte formation (11, 38). Due to the lack of early diagnostic indicators, OA patients often lose the best treatment opportunities, resulting in poor prognosis. Chronic inflammation of the synovial membrane of joint tissue is considered to be the most important cause of tissue damage, joint destruction, and disability (39). Therefore, identifying biomarker genes and their activation status in OA synovitis tissues is crucial to increasing our understanding of the underlying mechanisms and the identification of new therapeutic targets. With the rapid development of science and technology and the deepening understanding of the pathophysiology of OA, bioinformatics provides a powerful strategy for screening molecular markers and found a variety of therapeutic targets that may participate in the structural progress of OA, some of which are promising and under clinical investigation in randomized controlled trials (7). In this study, we attempted to identify diagnostic markers in synovitis of OA and their possible drug targets, and further explore the role and function of diagnostic markers in OA.

First, we downloaded the microarray expression profile data of 5 OA synovitis from GEO, and identified 3118 differentially expressed genes, among which seven overlapping genes coexist in five data sets. In order to exclude part of the false-positive markers, we used a LASSO machine learning method to build models and obtain biomarkers with higher accuracy. LASSO logistic regression determines variables by minimizing the classification error, which is mainly used to screen characteristic variables and build the optimal classification model. In this study, a total of six genes, TAC, CDKN1A, FOSB, STMN2, SLC2A3, and SCRG1, were identified as diagnostic markers of OA by feature selection using LASSO method, and a classification model of OA was constructed, whose validation set AUC was 0.9394. To further verify the reliability of the model’s classification and identification, two synovium-related RNA-SEQ datasets were used for validation. The results showed that the AUC of the validation set was 0.8713, indicating that the model is a very reliable diagnostic classifier; that six genes are feasible as biomarkers in OA. In this study, we found that the expression trend of three DEGs (CDKN1A, FOSB, SLC2A3) in the GSE82107 data set were inconsistent with other data sets from the common difference DEG analysis step. We considered that these three DEGs may have multiple regulatory functions in synovial inflammation, such as involvement in multiple cell reprogramming processes. We also observed different expression trends of these three DEGs in the validation RNA-seq datasets, therefore, more consideration is needed in the function research of these three DEGs. Similarly, we found that only three DEGs (SCRG1, CDKN1A, SLC2A3) had a significant P-value in the RNA-seq data validation step, and only the SCRG1 gene showed a consistent expression trend, which was increased in the OA synovitis samples, so SCRG1 may be a reliable biomarker and therapeutic target for OA diseases.

For the six marker genes previously identified, we found that CDKN1A, FOSB, STMN2, and SLC2A3 genes were reported to be associated with cartilage inflammation in OA (40–51). For example, the expression level of CDKN1A will increase correspondingly during cartilage degeneration (44), IL-17 and IL-1beta induced collagenase-3 production through AP-1 occurred with differential protein complex, and stimulation resulted in FOSB activation (52), STMN2 has significant differences between preserved and subchondral bone (48). SCRG1 has also been observed to be specifically expressed in human articular cartilage and is involved in human mesenchymal stem cell (hMSC) growth suppression and differentiation during dexamethasone dependent chondrogenesis (31). Ochi et al. found that overexpression of SCRG1 can inhibit the proliferation of hMSCs, stimulate cartilage formation in C3H10T1 cells, play a role in mesenchymal chondrogenesis *in vitro*, and may have an important function in cartilage development (31). So, we suggest that these six DEGs may be the key genes of OA. Moreover, related drug molecules were identified from six biomarkers according to DSigDB database in this study. Among all candidate drugs, VALPROIC ACID CTD 00006977, Tetradioxin CTD 00006848, estradiol CTD 00005920, benzopyrene CTD 00005488, and Retinoic acid CTD 00006918 ranked in the top five with targeting multiple DEGs (P-value<0.05). Previous studies have found that the Retinoic acid-related orphan receptor (RORα) has potential in mediating OA and changing the progression of OA (11, 28). The screened drug, VALPROIC ACID CTD 00006977, was a reverse agonist of RORα, suggesting that this drug may be a targeted therapy for OA disease. The efficacy of other proposed drugs is also speculated, and these drugs may be considered for further validation by chemical experiments.

Although all six genes are associated with cartilage, they are rarely reported in OA synovitis. In order to further explore the role of the six genes in OA synovitis, we used STRING database to construct the protein interaction network, but the results showed that SCRG1 did not exist in the constructed network. Combined with the above difference analysis results and the basic research results of SCRG1 gene in the literature, SCRG1 gene was significantly different in all microarray and RNA-seq data sets, and was upregulated in all analysis data sets, and the role and function of SCRG1 gene in OA synovitis was not clear; therefore, it is very important to study the function and possible mechanism of SCRG1 in OA synovitis. SCRG1 is a small protein with a length of 98 amino acids, rich in cysteine, is mainly distributed in the central nervous system (31), and was discovered through the identification of the genes associated with the neurodegenerative changes observed in transmissible spongiform encephalopathies (53). SCRG1 was originally named “Scrapie responsive gene-1” by Dr. Dron (32), and HGNC was later named “Stimulator of chondrogenesis 1”. The protein targets the Golgi apparatus and large dense-core vesicles and secretory granules of neurons (33). Recent studies have shown that SCRG1 is an important regulator during hMSC self-renewal, migration, and osteogenic differentiation along with its receptor BST1 (54), and has been found to be widely induced in neurons of scrapie-infected mice, suggesting that SCRG1 is involved in the host response to stress and neuronal death (33).

In order to explore the function of SCRG1 gene in OA, we obtained 27 genes consistent with the expression trend of SCRG1 gene by Spearman analysis in this study. When we annotated 27 gene functions using GO and KEGG databases, metabolism, complement, antigen presentation, apoptosis, and immune-related pathways were the most important functional category. Several studies have shown that dysfunctional synovial cell bioenergetics alter the distribution of OA synovial cells and promote inflammatory development. The protein network of 27 genes was constructed and the analysis network showed that the function of these genes was enriched in four aspects: immune response, cytokine production, migration, and osteoclast development. Immune response and cytokine production are directly related to inflammation. Interestingly, SCRG1 has been shown to be involved in immune regulation pathways in mouse cell lines (55), but the possible functional relationships between SCRG1 and neurons were not found, which might be a pitfall of bioinformatics or AI learning. Analyses by Dron’s group revealed colocalization of SCRG1 with SCG2, TAC2 (34), GM130, and protein disulfide isomerase (33). We suggest that SCRG1 may be involved in neuro-related biological functions, although colocalizations of SCRG1 with other molecules were detected by immunohistochemical analyses and may not be interpreted as evidence of protein-protein interactions, but there is still reason to believe that SCRG1 may be involved in neurobiological function (35). On the other hand, when we analyzed the genes interacting with SCRG1, we did not find the expression of the BST1 gene associated with the SCRG1 ligand. We suggest that BST1 might be communicating with specific cell types in OA synovitis by cell communication. While the data used in the analysis were not representative of BST1 gene expression in specific cell types of OA synovitis, the single-cell sequencing may help us to understand the role of SCRG1 in cell communication. Meanwhile, since forced expression of SCRG1 in hMSCs will be suppressed cell proliferation and stimulated chondrogenesis (31), and combined with the previous results obtained from synovial data we used, we believe that the role of the SCRG1 gene in OA synovitis may be related to the pathogenesis of disease, rather than the result of enhanced chondrogenesis to compensate for primary abnormalities such as degeneration of cartilages.

Further studies on the TFs-miRNA co-regulatory network found that hsa-mir-363-3p may be significantly regulated by SCRG1, which results in the miRNA dataset (39), indicating that the function of SCRG1 in OA may be regulated by miRNA hsa-mir-363-3p. It also revealed the important contribution of miRNA to transcriptome changes in OA synovial cells and explained the complexity of mutual regulation between miRNA and mRNA. Although several miRNA targets (hsa-mir-92b-5p, hsa-mir-32-3p, hsa-mir-92b-3p, and hsa-mir-25b-3p) were not validated with published data, important evidence of interactions between these targets and corresponding miRNAs was found from mirTarBase, and these targets should be followed up in future studies. Furthermore, considering the therapeutic potential of miRNAs in preclinical studies of OA, miRNA interaction for this dysfunction may be promising for OA.

The absence of experimental validation of biological samples is a significant limitation of this study, although the microarray and RNA-seq methods were combined to reduce the deviation introduced by using a single method, and RNA-seq data were also used to validate the conclusions. However, the sample data used still do not fully reflect all aspects of the transcriptome characteristics of OA synovitis. On the other hand, it should be mentioned that the validation RNA-seq datasets are weighted heavily towards GSE89408 because of clinical sample sizes. In this study, GSE89408 dataset contained 22 OA and 28 normal samples while GSE143514 contained only five OA and three normal samples were included in the validation RNA-seq datasets. In general, enlarging the sample size, adding tissue-specific data, and verifying the biological function will effectively prove the conclusion of the analysis.

In summary, this study combined bioinformatics and machine learning methods to analyze the transcriptional expression characteristics of OA synovitis and screened six biomarkers related to OA. Drug database enrichment found that these six DEGs may be the drug targets of synovitis in osteoarthritis, and Valproic Acid CTD 00006977 may be a potential targeted therapeutic drug of SCRG1. SCRG1 is upregulated in OA synovitis and is manifested by the increased immune response, suggesting that SCRG1 is involved in cell growth suppression and differentiation. miRNA hsa-miR-363-3p plays an important role in regulating SCRG1 in OA synovitis. The six genes identified in this study can provide potential targets for the diagnosis and treatment of OA. As a reliable biomarker and therapeutic target for OA synovitis, the extent to which SCRG1 upregulated promotes the development of OA in synovitis and the corresponding function remains to be studied.

## Conclusions

SCRG1, as a diagnostic marker of OA, may be significantly up-regulated by hsa-miR-363-3p in synovitis of OA, and co-regulate immune-related pathways through the interaction of related proteins, playing an important role in the occurrence and development of OA, which may be a new drug target.

## Data Availability Statement

Publicly available datasets (GSE1919, GSE12021, GSE55235, GSE55457, GSE82107, GSE89408 and GSE143514) were analyzed in this study. All the datasets were obtained from the GEO (http://www.ncbi.nlm.nih.gov/geo) database.

## Author Contributions

GL analyzed and wrote the manuscript. GH designed the experiments and analyzed the data. ZZ devised the concept and supervised the study. All authors contributed to the article and approved the submitted version.

## Conflict of Interest

The authors declare that the research was conducted in the absence of any commercial or financial relationships that could be construed as a potential conflict of interest.

## Publisher’s Note

All claims expressed in this article are solely those of the authors and do not necessarily represent those of their affiliated organizations, or those of the publisher, the editors and the reviewers. Any product that may be evaluated in this article, or claim that may be made by its manufacturer, is not guaranteed or endorsed by the publisher.

## Supplementary Material

Supplementary Figure 1 GO functional analysis of 27 related genes associated with SCRG1. **(A-C)** The Go analysis result showed by biology process, cellular component, molecular function. The results showed that 27 genes function was associated with immune signaling pathway, may be involved in synovial inflammation.
