# Supplementary figures and images for: Identification of SCRG1 as a Potential Therapeutic Target for Human Synovial Inflammation
Source: Front Immunol. 2022 May 26;13:893301. doi: 10.3389/fimmu.2022.893301 (PMC9204521; doi:10.3389/fimmu.2022.893301)

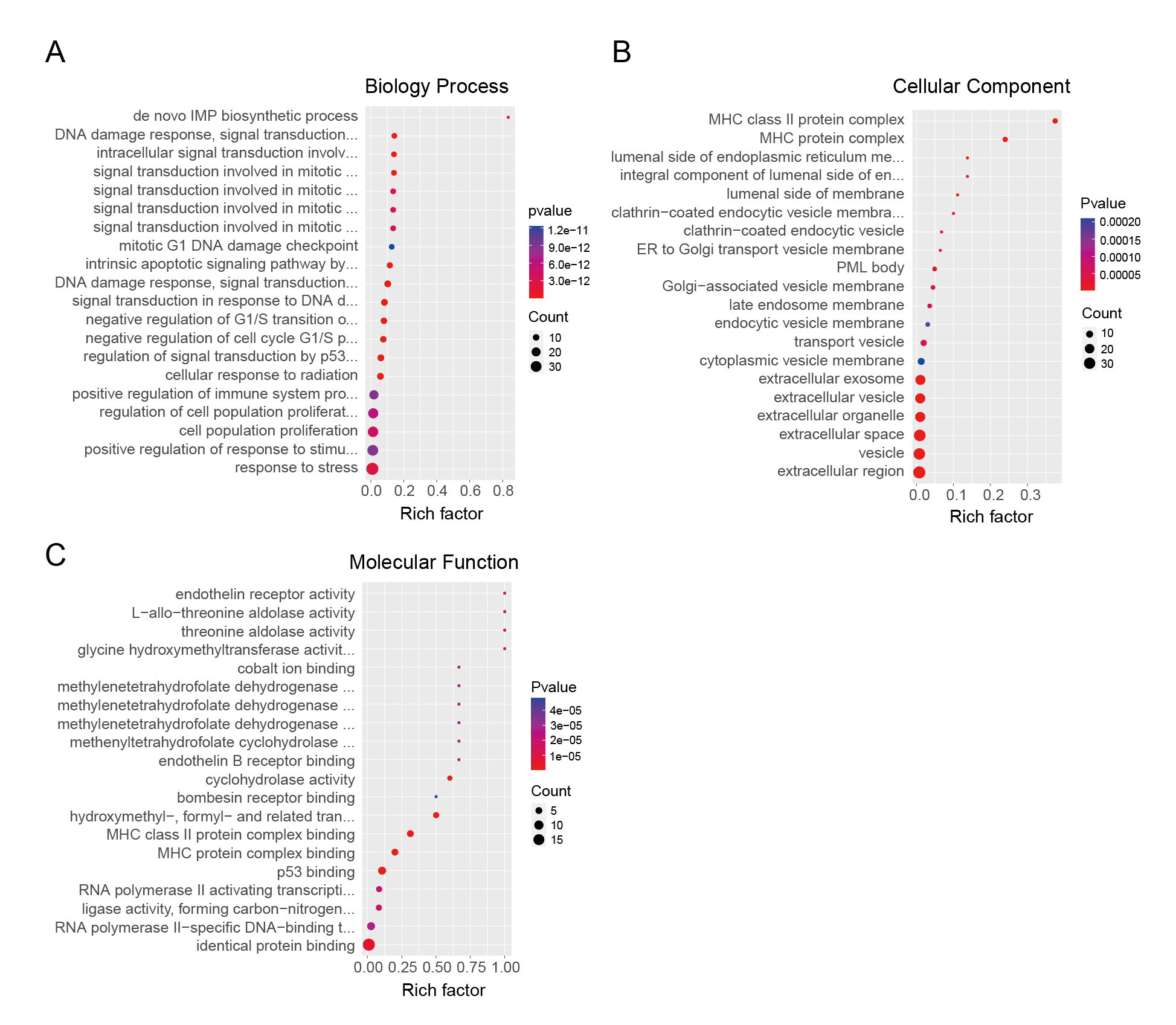

Supplement: Supplementary Figure 1 — GO functional analysis of 27 related genes associated with SCRG1. (A-C) The Go analysis result showed by biology process, cellular component, molecular function. The results showed that 27 genes function was associated with immune signaling pathway, may be involved in synovial inflammation. [file Image_1.jpeg]
